# Precise visualization and ROS-dependent photodynamic therapy of colorectal cancer with a novel mitochondrial viscosity photosensitive fluorescent probe

**DOI:** 10.1186/s40824-023-00450-2

**Published:** 2023-11-08

**Authors:** Runsha Xiao, Fan Zheng, Kuo Kang, Lei Xiao, Anyao Bi, Yiting Chen, Qi Zhou, Xueping Feng, Zhikang Chen, Hao Yin, Wei Wang, Zihua Chen, Xiaomiao Cheng, Wenbin Zeng

**Affiliations:** 1 Department of General Surgery, Xiangya Hospital, Central South University, Changsha, 410013 People’s Republic of China; 2 Hunan Key Laboratory of Precise Diagnosis and Treatment of Gastrointestinal Tumor, Xiangya Hospital, Central South University, Changsha, 410013 People’s Republic of China; 3 National Clinical Research Center for Geriatric Disorders, Xiangya Hospital, Central South University, 410013 Changsha, People’s Republic of China; 4 Xiangya School of Pharmaceutical Sciences, Central South University, Changsha, 410013 People’s Republic of China; 5 Hunan Key Laboratory of Diagnostic and Therapeutic Drug Research for Chronic Diseases, Central South University, Changsha, 410013 People’s Republic of China; 6 Department of Colorectal Surgery, Affiliated Cancer Hospital of Xiangya School of Medicine, Central South University, Changsha, 410013 People’s Republic of China; 7 Department of Critical Care Medicine, Xiangya Hospital, Central South University, Changsha, 410013 People’s Republic of China; 8 Institute of Medical Sciences, Xiangya Hospital, Central South University, Changsha, 410013 People’s Republic of China; 9 Organ Transplant Center, Shanghai Changzheng Hospital, Shanghai, 200003 People’s Republic of China; 10 Cell Transplantation and Gene Therapy Institute, The Third Xiang Ya Hospital, Central South University, Changsha, 410013 People’s Republic of China; 11 Engineering and Technology Research Center for Xenotransplantation of Hunan Province, Changsha, 410013 People’s Republic of China; 12 Department of Nephrology, Xiangya Hospital, Central South University, Changsha, 410013 People’s Republic of China; 13 Department of Nephrology, Xiangya Changde Hospital, Changde, 415000 People’s Republic of China

**Keywords:** Colorectal cancer, Fluorescent probe, Precise visualization, Viscosity, Mitochondrion-specific photosensitizer, Photodynamic therapy, Theranostics

## Abstract

**Background:**

Colorectal cancer (CRC) is a prominent global cancer with high mortality rates among human beings. Efficient diagnosis and treatment have always been a challenge for CRC management. Fluorescence guided cancer therapy, which combines diagnosis with therapy into one platform, has brought a new chance for achieving precise cancer theranostics. Among this, photosensitizers, applied in photodynamic therapy (PDT), given the integration of real-time imaging capacity and efficacious treatment feasibility, show great potential to serve as remarkable tools. Although much effort has been put into constructing photosensitizers for locating and destroying CRC cells, it is still in high need to develop novel photosensitizers to attain specific detection and fulfil effective therapy.

**Methods:**

Probe HTI was rational synthesized for the diagnosis and treatment of CRC. Spectrometric determination was carried out first, followed by the ^1^O_2_ generation ability test. Then, HTI was displayed in distinguishing CRC cells from normal cells Further, the PDT effect of the photosensitizer was studied in vitro. Additionally, HTI was used in CRC BALB/c nude mice model to validate its viscosity labelling and tumor suppression characteristics.

**Results:**

We successfully fabricated a mitochondrial targeting probe, HTI, together with remarkable viscosity sensitivity, ultralow background interference, and excellent ^1^O_2_ generation capacity. HTI was favorably applied to the viscosity detection, displaying a 11-fold fluorescent intensity enhancement in solvents from 1.57 cp to 2043 cp. Then, it was demonstrated that HTI could distinguish CRC cells from normal cells upon the difference in mitochondrial viscosity. Moreover, HTI was qualified for producing ^1^O_2_ with high efficiency in cells, supported by the sparkling signals of DCFH after incubation with HTI under light irradiation. More importantly, the viscosity labelling and tumor suppression performance in CRC CDX model was determined, enriching the multifunctional validation of HTI in vivo.

**Conclusions:**

In this study, HTI was demonstrated to show a sensitive response to mitochondrial viscosity and possess a high ^1^O_2_ generation capacity. Both in vitro cell imaging and in vivo tumor treatment trials proved that HTI was effectively served as a robust scaffold for tumor labeling and CRC cells clearance. This breakthrough discovery held immense potential for advancing the early diagnosis and management of CRC through PDT. By leveraging HTI's properties, medical professionals could benefit from improved diagnostic accuracy and targeted treatment in CRC management, ultimately leading to enhanced patient outcomes.

**Supplementary Information:**

The online version contains supplementary material available at 10.1186/s40824-023-00450-2.

## Introduction

Colorectal cancer (CRC), the second most common cancer in global prevalence and mortality, induces over 5.3 million prevalent cases and 0.9 million deaths in five years [1, 2]. It is ascribed to the unhealthy lifestyle and the worsening environmental contamination that leads to continuous growth of CRC, presenting a significant threat to human health [3–5]. In this scenario, numerous resources are allocated towards biomedical research aimed at advancing the progress of novel diagnostic techniques and efficacious drugs for cancer [6–10]. So far, imaging modalities, such as positron emission tomography (PET), computed tomography (CT), and magnetic resonance imaging (MRI), are typically involved in the early diagnoses of CRC [11, 12]. Nevertheless, these methods generally suffer from long acquisition time, as well as expensive cost [13, 14]. In this predicament, fluorescence imaging, in view of its fast responsive capability, high sensitivity, and low toxicity, has attracted much attention [15, 16].

Inspired by the merit of fluorescence imaging, fluorescence guided cancer therapy, which combines diagnosis with therapy into one platform, has brought a new chance for achieving precise cancer theranostics [17–19]. Among this, given the inherent fluorescence of the photosensitizer used in photodynamic therapy (PDT), photosensitizers show great potential to serve as remarkable tools to realize fluorescence guided cancer therapy [20, 21]. PDT is a talented noninvasive cancer therapeutic modality in which photosensitizers can generate cytotoxic reactive oxygen species (ROS) upon light irradiation, leading to cancerous cells death (Table S1) [22–24]. Meanwhile, based on the broad application of endoscopy, it makes photosensitizers applicable for the identification and treatment of malignant lesions in CRC, which can minimize the effect of the limited penetration depth of light on the theranostic efficiency [25–27]. Although much effort has been devoted into constructing photosensitizers for locating and destroying CRC cells, it is still in high need to develop novel photosensitizers with excellent tissue selectivity to attain specific detection, as well as superior PDT efficacy to fulfil effective therapy.

Since the occurrence and development of cancer is closely associated with tumor microenvironment, monitoring the fluctuation in it can be a potential direction for cancer diagnosis [28]. Typically, viscosity, as an essential microenvironmental parameter, can affect the diffusion and interaction processes of the intracellular substances [29, 30]. The abnormal changes in viscosity might result in malignant growth of cells [31, 32]. Meanwhile, the unique proliferation of cancer cells will cause the accumulation of lactic acid, further facilitating the display of higher viscosity in tumor cells compared to normal cells [16, 33]. Thus, it is of great significance to develop probes to differentiate intracellular viscosity for tumor-specific imaging. On the other hand, the efficacy of PDT is greatly hindered by the finite ROS production capacity of photosensitizers and the short diffusion distance of ROS [34–36]. Considering those mitochondrial crucial roles in multifarious metabolic processes, such as cell growth, energy renewal, and cell apoptosis, direct targeting to mitochondria can theoretically maximize the photodynamic effect of the photosensitizers [37–40]. As such, the development of mitochondria-specific photosensitizers has captured sustained attention. Consequently, the rational design of photosensitizers, with viscous sensitivity and mitochondrial targetability, has been arising as a promising way for efficient CRC theranostics.

**Scheme 1:**
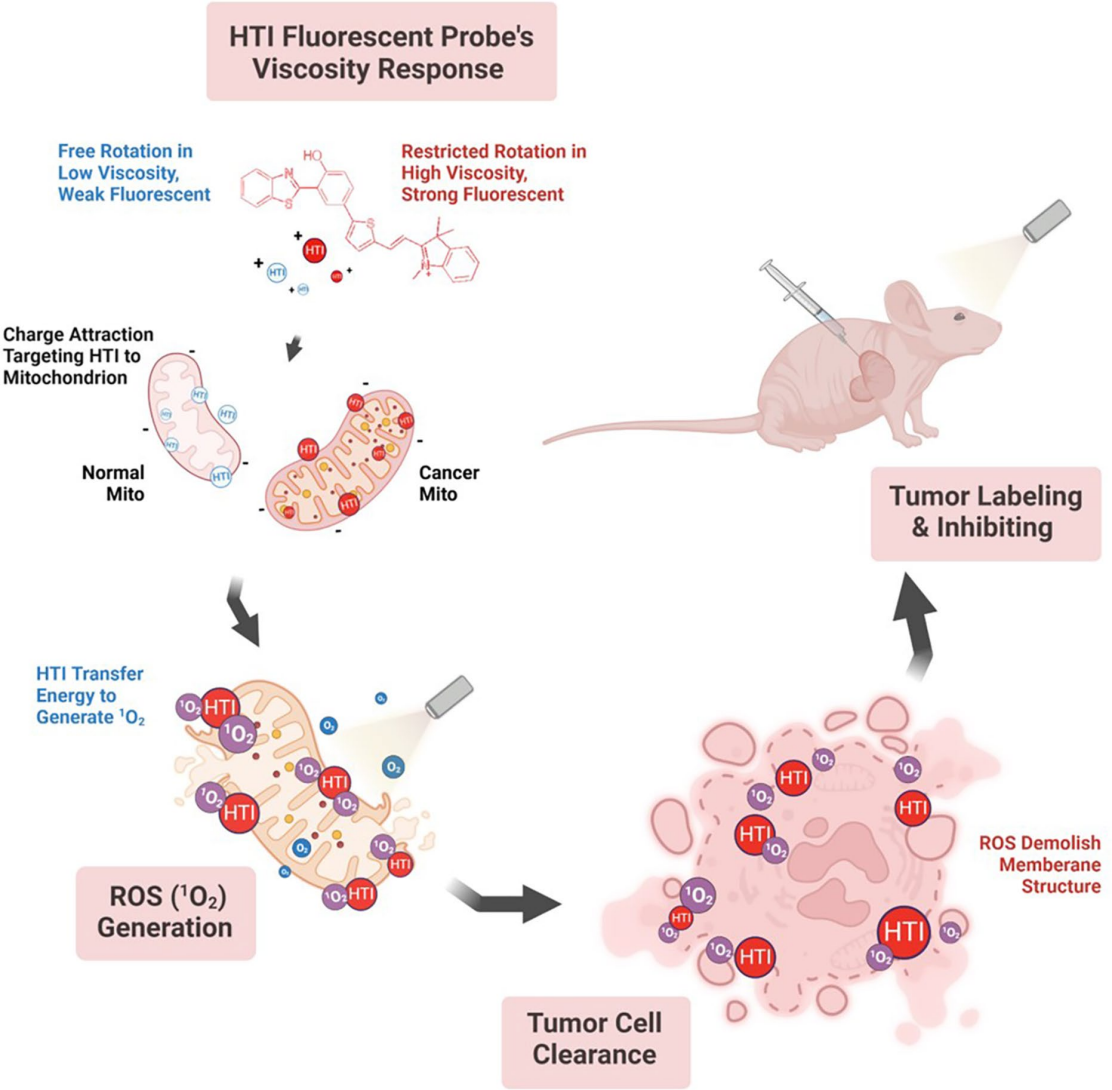
Schematic illustration of HTI for precise visualization and ROS-dependent photodynamic therapy of colorectal cancer. Created with BioRender.com

Herein, we reported the development of a novel photosensitizer, 2-(2-(5-(3-(benzo[d]thiazol-2-yl)-4-hydroxyphenyl)thiophen-2-yl)vinyl)-1,3,3-trimethyl-3H-indol-1-ium iodide (HTI), benefiting from its remarkable viscosity sensitivity, small background interference, and excellent mitochondria-targeting ability, for the diagnosis and treatment of CRC. As illustrated in Scheme 1, the free intramolecular rotation of HTI could gradually be restricted by increased viscosity which led to enhanced fluorescence intensity of the probe. With a main accumulation in mitochondria, HTI was then utilized for monitoring the intracellular changes in viscosity. Moreover, the probe could distinguish CRC cells from epithelial cells based on the differences in the microenvironmental viscosity and the mitochondrial membrane potential. More importantly, the high singlet oxygen (^1^O_2_) generation capacity of the probe enabled it to effectively inhibit the growth of colon tumor. In summary, HTI was considered as a potential fluorescence imaging-based theranostic probe for CRC with real-time visualization capabilities and high PDT efficiency.

## Results

### Design and synthesis of HTI

In this work, we have successfully developed a viscosity-sensitive and mitochondria-targeting fluorescent probe HTI on basis of a rational molecular design strategy (Scheme S1). It was synthesized through the conjugated connection of the electron donor 2-(2’-hydroxyphenyl)benzothiazole with the electron acceptor 2,3-dimethylbenzo[d]thiazol-3-ium iodide by thiophene as a π bridge. The fluorescence of HTI was nearly quenched in low viscous media since the probe would form a twisted intramolecular charge transfer state upon excitation. In contrast, the free rotation in HTI would be restricted in a higher-viscosity system, leading to a significant fluorescence enhancement. Moreover, the strong push–pull effect and the finely tuned intramolecular rotation enabled the excited energy of the probe to be vulnerably released through nonradiative relaxation, revealing the potential use of HTI as a photosensitizer for PDT. Thus, HTI was applicable for displaying both diagnostic and therapeutic performances for cancer. NMR and HRMS analysis of the probe were presented in the supporting information (Fig. S1-S3).

### Spectroscopic properties of HTI

**Fig. 1:**
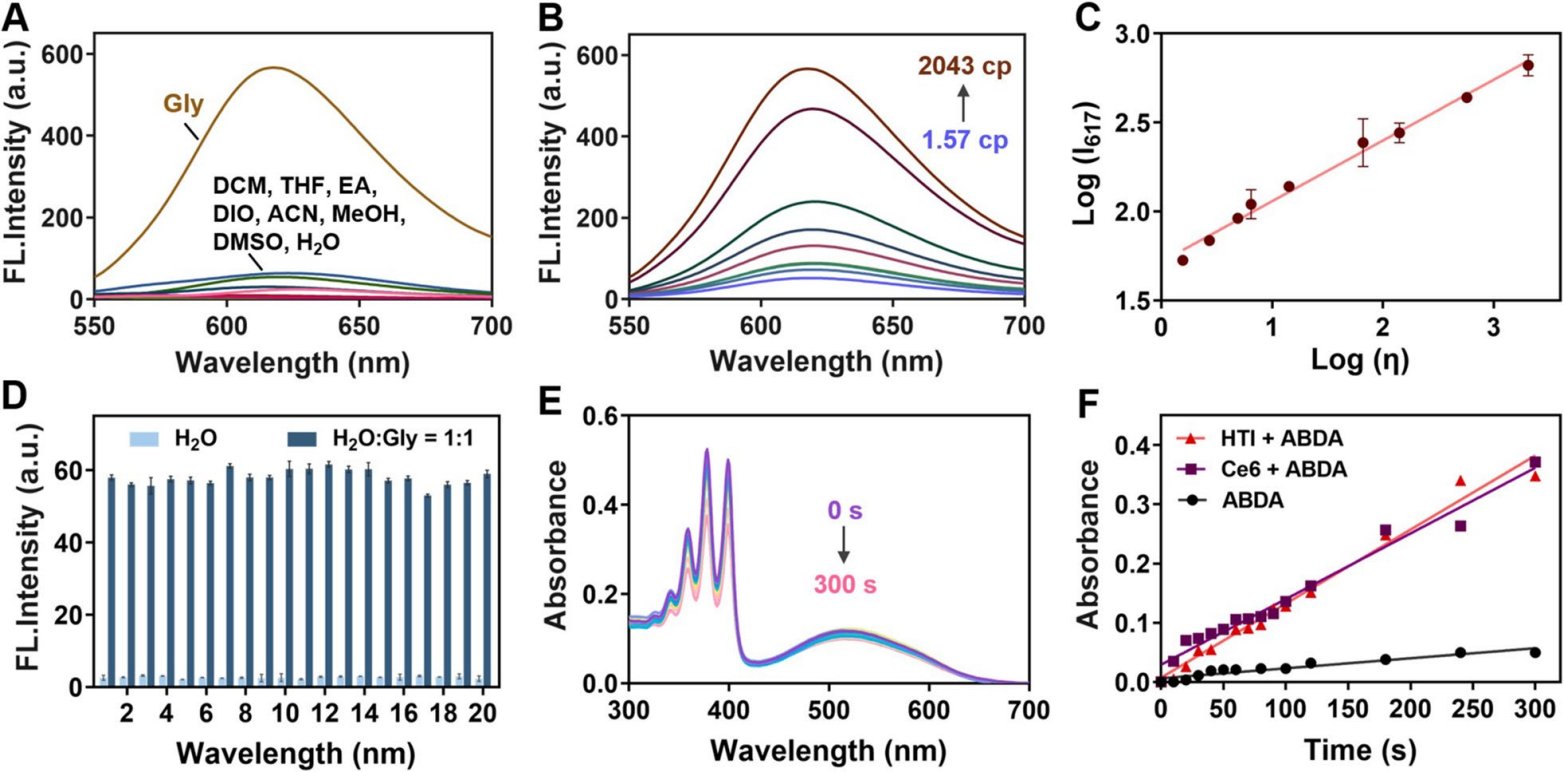
**A** Fluorescence spectra of HTI (10 μM) in water and various organic solvents. **B** Fluorescence spectra of HTI (10 μM) in mixed solvents with different proportions of methanol and glycerin. **C** Dependence of Log I on Log η. Log(I_617_) = 0.3419 log η + 1.709 (R^2^ = 0.9935). **D** Fluorescence responses of HTI (10 μM) for various analytes in H_2_O or H_2_O/Gly (1/1, v/v). Analytes (HTI, 10 μM; other analytes, 100 μM): 1, Only probe; 2, Zn(OAc)_2_; 3, FeSO_4_; 4, CoCl_2_; 5, CaCl_2_; 6, MgSO_4_; 7, NaF; 8, NaBr; 9, NaI; 10, KO_3_; 11, NaNO_2_; 12, Na_2_SO_3_; 13, H_2_O_2_; 14, Glucose; 15, Cys; 16, Tyr; 17, Pro; 18, Gly; 19, Val; 20, Ala. *λ*_ex_ = 500 nm. **E** UV–vis absorption spectra of ABDA (50 μM) with HTI (10 μM) upon various periods of white light irradiation (60 mW cm^−2^). **F** Changes of absorption intensity of ABDA solution at 378 nm upon irradiation for different times

First, the absorption of HTI was investigated. As shown in Fig. S4, an obvious absorbance band of HTI was located at around 500 nm. Compared to that in methanol (501 nm, ε = 1.78 × 10^4^ M^−1^ cm^−1^), the absorption peak red-shifted in glycerin (511 nm, ε = 1.37 × 10^4^ M^−1^ cm^−1^). Next, the emission spectra of the probe were carried out in water and diverse organic solvents. As can be seen from Fig. 1A, distinct fluorescence was observed only in glycerin, indicating the response of HTI to viscosity rather than to polarity. Subsequently, the quantitative evaluation against viscosity was provided in mixed solvents with different proportions of methanol and glycerin. As depicted in Fig. 1B, the fluorescence intensity at 617 nm increased with the ratio of glycerin which ultimately raised 11-fold from 1.57 cp to 2043 cp. Remarkably, good linearity was displayed between log I_617_ and log η based on a Förster − Hoffmann equation of log I_617_ = 0.3419 log η + 1.709 (Fig. 1C). These results implied that HTI possessed a high sensitivity to viscosity. Then, the specificity of the probe to viscosity was demonstrated in comparison with other biological analytes, such as Zn^2+^, Ca^2+^, NO_2_^−^, SO_3_^2−^, Cys, and Ala (Fig. 1D). Meanwhile, few fluctuations in fluorescence were exhibited in phosphate buffer saline (PBS) with different pH (Fig. S5). These results integrally confirmed that HTI was feasible for visualizing viscous changes.

### ROS generation ability of HTI

Emerging anticancer strategies were devoting to advancing targeting ability and precision towards CRC. Among them, analyzing the characteristic signals of CRC, such as acid, β-glucuronidase and hydrogen sulfide [41–43], had proffered a new approach to realize precise identification and bundled delivery of therapeutic groups. PDT, a promising anti-cancer method, could induce the generation of ROS under light irradiation to trigger cancer cell apoptosis. However, the low efficacy caused by uncertain subcellular localization remained a challenge. Thus, we conducted trials to validate HTI’s oncotherapy performance in vitro and in vivo. To begin with, we compared the ^1^O_2_ generation capacity of HTI towards a commercial clinical photosensitizer, Ce6, via a ^1^O_2_ indicator ABDA (Fig. 1E and Fig. S6). As shown in Fig. 1F, the slope of the HTI group was larger than that of the Ce6 group, indicating a stronger ^1^O_2_ generation capacity of HTI under white light (60 mW cm^−2^) irradiation.

### Monitoring the variations of mitochondrial viscosity in living cells

Given the favorable properties of HTI in vitro, we investigated the cytotoxicity of HTI probe in colon cell lines, including HCT-116, SW-620, and NCM-460 cells via CCK8 assay before inspecting the performance of HTI at the subcellular levels. Apparently, the survival of these colon cell lines still firmly maintained over 87% at concentrations up to 20 μM of HTI (Fig. S7). The results supported that HTI was a biosafe scaffold, and the optimal application concentration for the subsequent experiments was assessed in view of this.

**Fig. 2:**
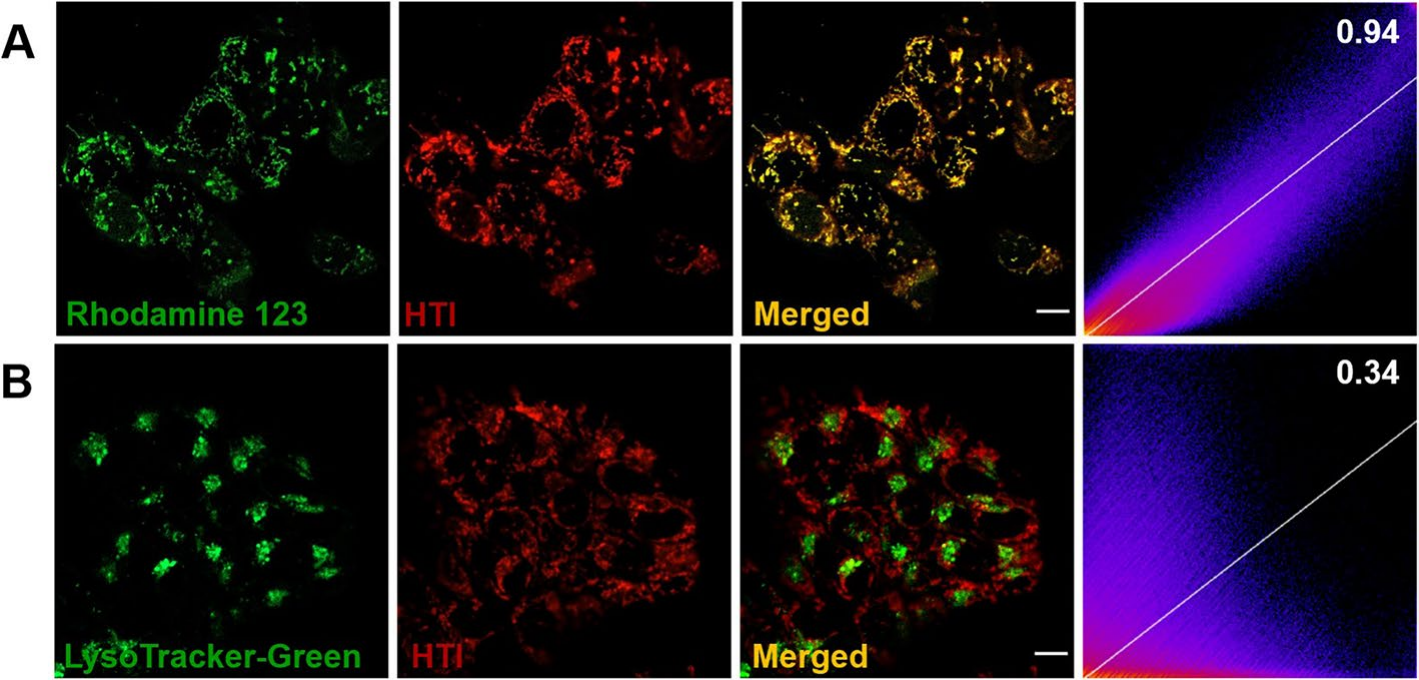
Colocalization images of HTI (10 μM) co-stained with Rhodamine 123 (**A**) or LysoTracke-Green (**B**) in HCT-116 cells. Green channel for Rhodamine 123 and LysoTracker-Green: *λ*_ex_ = 488 nm, *λ*_em_ = 525 ± 20 nm. Red channel for HTI: *λ*_ex_ = 570 nm, *λ*_em_ = 610 ± 20 nm. Scale bar, 10 μm

Then, the co-localization imaging using CLSM was conducted to elucidate the targeting capability of HTI (Fig. 2 and Fig. S8). HTI was co-stained with Mitotracker Rhodamine 123 or Lysotracker Green in HCT-116 cells, respectively. As expected, the reticular mitochondrial filamentous structure widely distributed in the cytoplasm was captured in the green channel of Rhodamine 123. Meanwhile, it was precisely overlapped with the red fluorescence of HTI with a Pearson’s correlation coefficient (PC) up to 0.94 (Fig. 2A). Relatively, as depicted in Fig. 2B, the red channel signal of HTI was distinctly distinguished from the green fluorescence of Lysotracker Green with clear boundaries (PC as 0.34). These results indicated HTI was capable to achieve mitochondrial directing, laying a foundation for subsequent functional implementation.

**Fig. 3:**
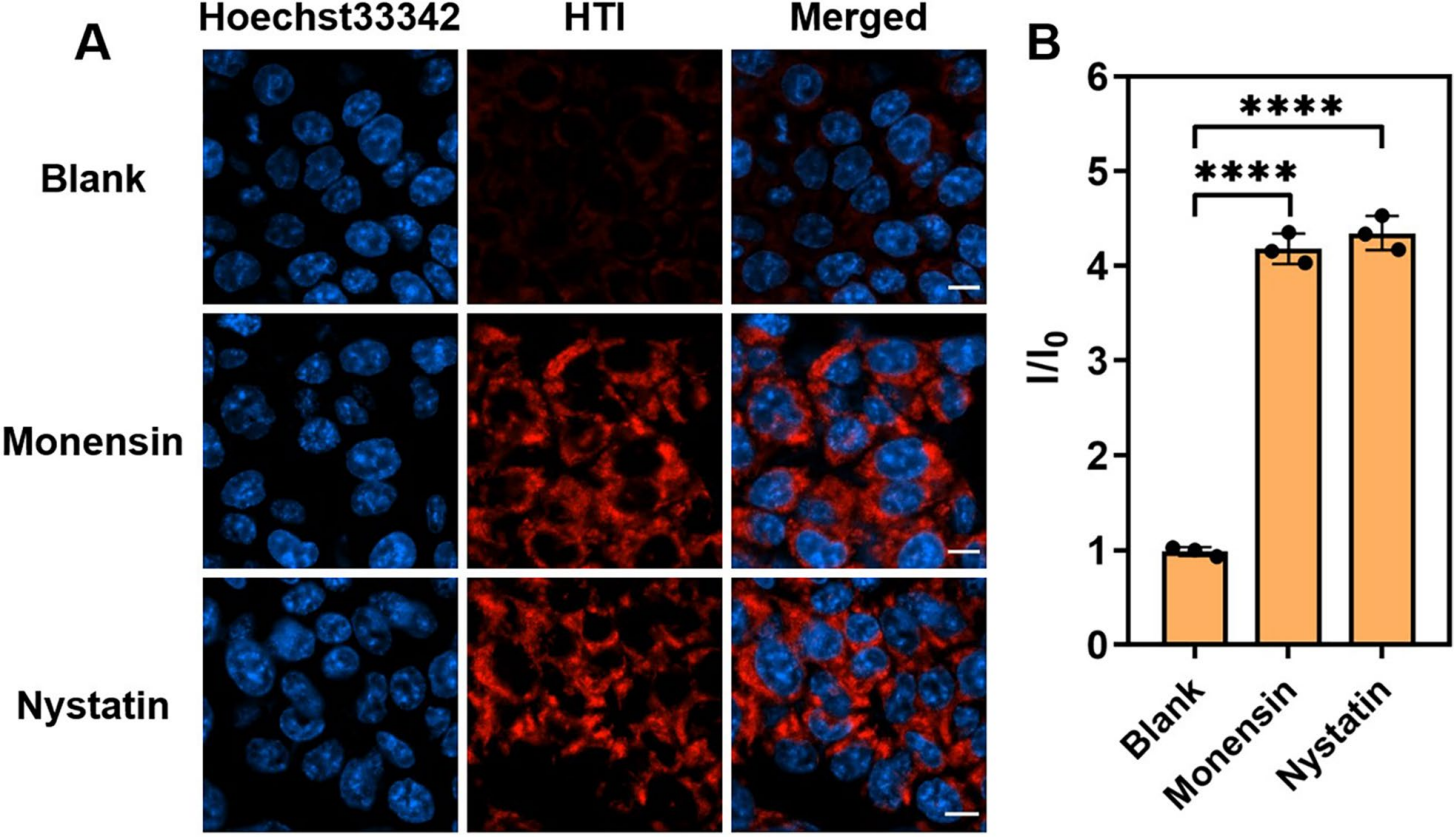
Viscosity detection in HCT-116 cells with HTI (10 μM) under different stimulants. **A** CLSM images of cells incubated with HTI for 30 min in different viscosity stimulants, monensin and nystatin, separately. Blue channel for Hoechst-33342: *λ*_ex_ = 405 nm, *λ*_em_ = 455 ± 20 nm. Red channel for HTI: *λ*_ex_ = 570 nm, *λ*_em_ = 610 ± 20 nm. Scale bar, 10 μm. **B** The plots of relative fluorescence intensities of the blank group vs the monensin group vs the nystatin group. Data are presented as mean ± s.d. (*n* = 3). **** *p* < 0.0001

Inspired by the outstanding visualizing potential towards mitochondria, the efficiency of HTI in monitoring the intracellular fluctuations of viscosity was then assessed. As reported, monensin and nystatin could induce mitochondrial viscosity raising by causing mitochondrial structure changes and swelling [44]. They were used as viscosity inducers to assess HTI’s viscosity visualization of mitochondria in HCT-116 cells (Fig. S9). As illustrated in Fig. 3, the monensin group and the nystatin group were observed to act a higher fluorescence intensity than the blank group. The results revealed that HTI was capable of real-time and in situ monitoring mitochondrial viscosity in live cells.

### Distinguishing CRC cells from normal cells

**Fig. 4:**
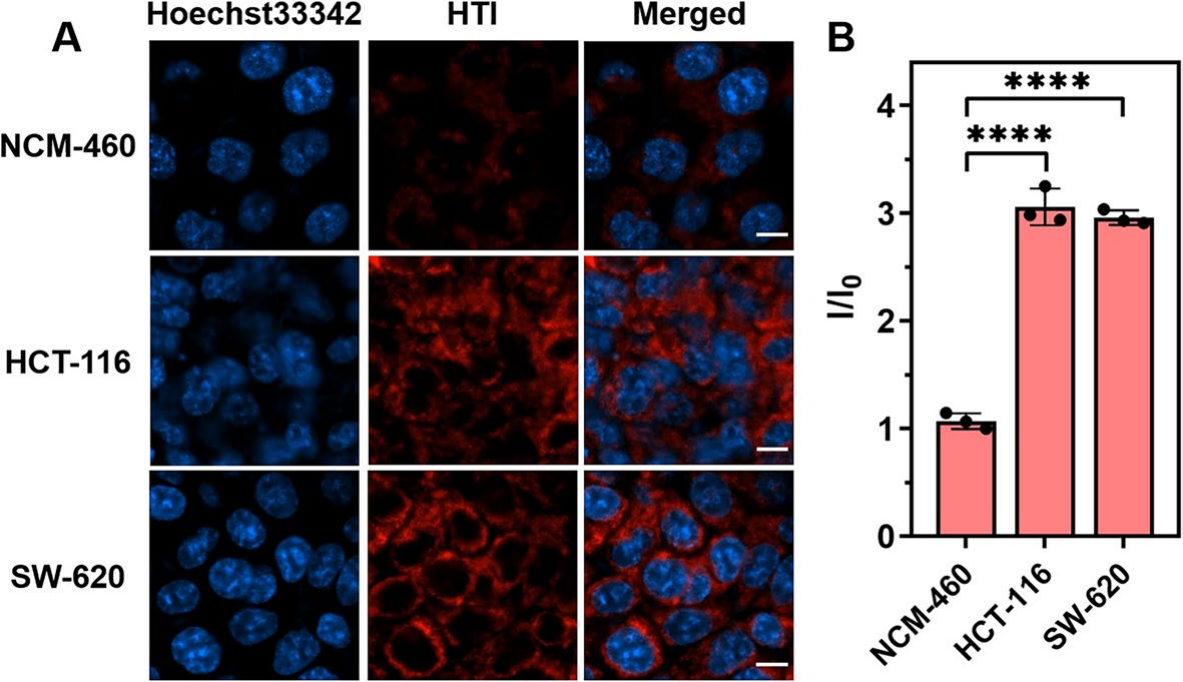
Distinguishing CRC cells from normal cells. **A** Viscosity inspection using HTI (10 μM) in epithelial cells (NCM-460) and in different colon cells (HCT-116 and SW-620). Blue channel for Hoechst33342: *λ*_ex_ = 405 nm, *λ*_em_ = 455 ± 20 nm. Red channel for HTI: *λ*_ex_ = 570 nm, *λ*_em_ = 610 ± 20 nm. Scale bar, 10 μm. **B** The plots of relative fluorescence intensities of NCM-460 vs HCT-116 vs SW-620, separately. Data are presented as mean ± s.d. (*n* = 3). **** *p* < 0.0001

Precise identification of tumor cells is of great significance for tumor management. Fluorescent probes are gaining popularity in the application of tumor pathophysiological environment detection due to its merits of low cost, high sensitivity, and real-time monitoring [45, 46]. Therefore, HTI was applied to compare the mitochondrial viscosity between human normal colon epithelial cells (NCM-460) and human colon cancer cells (HCT-116, SW-620) after fully verifying the viscosity inspecting characteristic. As depicted in Fig. 4, the red fluorescence of HTI in the colon cancer cells showed significant differences in comparison with that in the normal colon epithelial cells. These results implied that the mitochondrial viscosity of CRC cells displayed distinct differences from normal cells monitored by HTI. The specificity of HTI in CRC cells could lead the probe to providing an important and effective tool for clinical screening of CRC.

### ROS activation and PDT effect

**Fig. 5:**
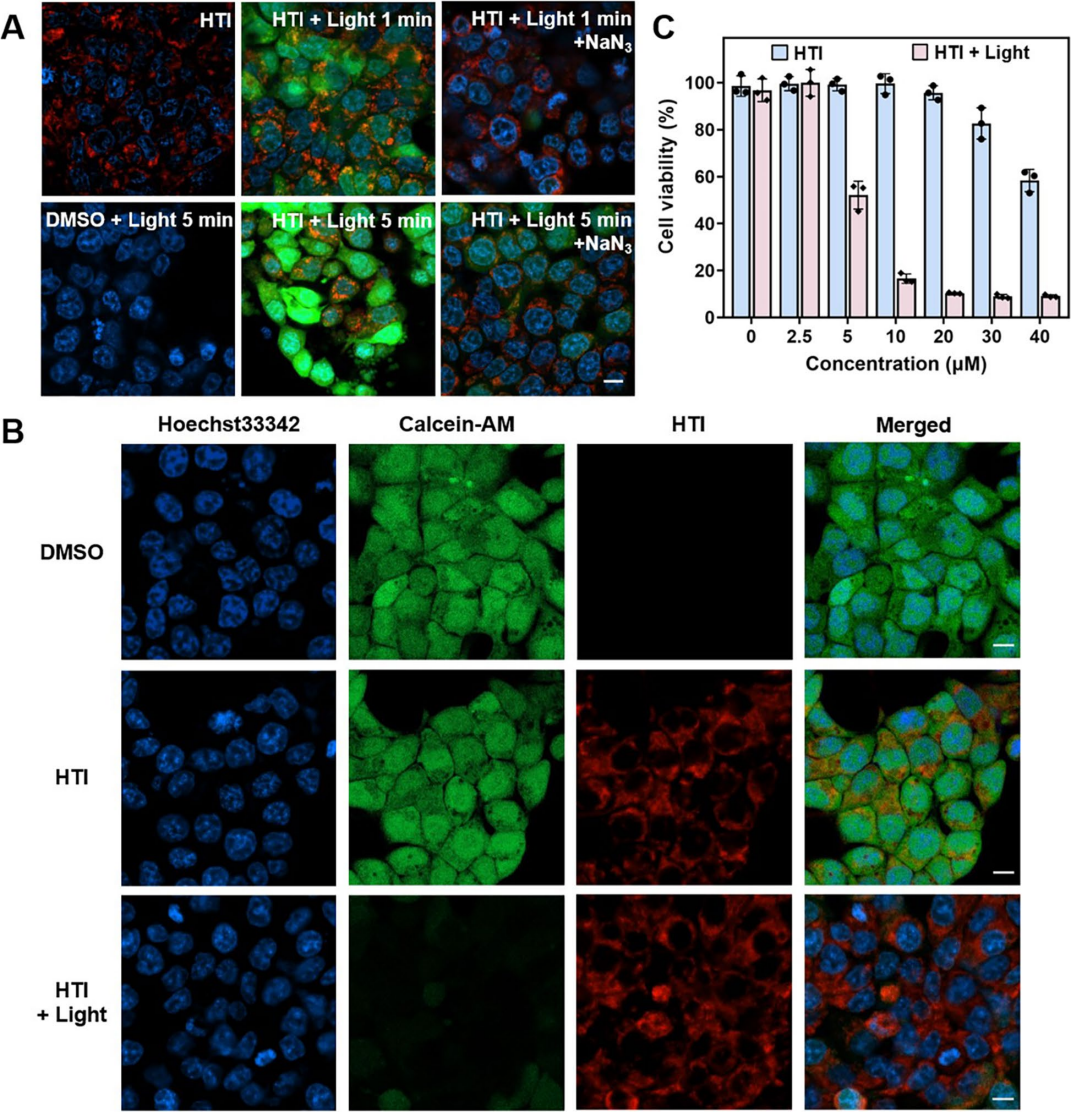
ROS generation and tumor cell clearance ability in HTI-labelled HCT-116 cells. **A** CLSM images depicted the increasing amount of ROS stimulated by HTI under 1 min or 5 min white light irradiation. Blue channel for Hoechst33342: *λ*_ex_ = 405 nm, *λ*_em_ = 455 ± 20 nm. Green channel for DCFH-DA: *λ*_ex_ = 488 nm, *λ*_em_ = 525 ± 20 nm. Red channel for HTI: *λ*_ex_ = 570 nm, *λ*_em_ = 610 ± 20 nm. Scale bar, 10 μm. **B** CLSM images illustrated the tumor cell clearance ability of HTI under white light irradiation. Blue channel for Hoechst33342: *λ*_ex_ = 405 nm, *λ*_em_ = 455 ± 20 nm. Green channel for Calcein-AM: *λ*_ex_ = 488 nm, *λ*_em_ = 525 ± 20 nm. Red channel for HTI: *λ*_ex_ = 570 nm, *λ*_em_ = 610 ± 20 nm. Scale bar, 10 μm. **C** The plot of cell viability of HCT-116 cells incubated with HTI at different concentrations with or without white light irradiation, respectively

ROS generation ability can decisively determine the PDT performance of a photosensitizer [47]. The ROS levels and the therapeutic effects were lucubrated at the cellular level. Utilizing DCFH-DA as a ROS indicator and NaN_3_ as a ^1^O_2_ scavenger, different irradiation times were applied to investigated the ROS generating procedure in HTI-incubated HCT-116 cells. As illustrated in Fig. 5A, the fluorescence of DCFH increased with the irradiation time, revealing HTI as a controllable photosensitizer in practical application scenarios. Subsequently, the therapeutic effects were explored by Calcein-AM and Hoechst-33342. Nucleus of both dead and live cells could be imaged in blue fluorescence by Hoechst-33342, whereas Calcein-AM could only provide green fluorescence in the cytoplasm of live cells. Based on this, since the green fluorescence extremely decreased after irradiation of the HTI-treated cells, it reflected that the cell viability immensely reduced after the incubation with HTI under irradiation, implying the high potential of HTI to effectively eliminate cancer cells. Furthermore, the cell viability was concisely elucidated through a CCK8 assay. As can be seen from Fig. 5C, there was a definitive dose-dependent and laser power density-dependent cytotoxicity. Overall, these results manifested that HTI exhibited high cytotoxicity against cancer cells through PDT.

**Fig. 6:**
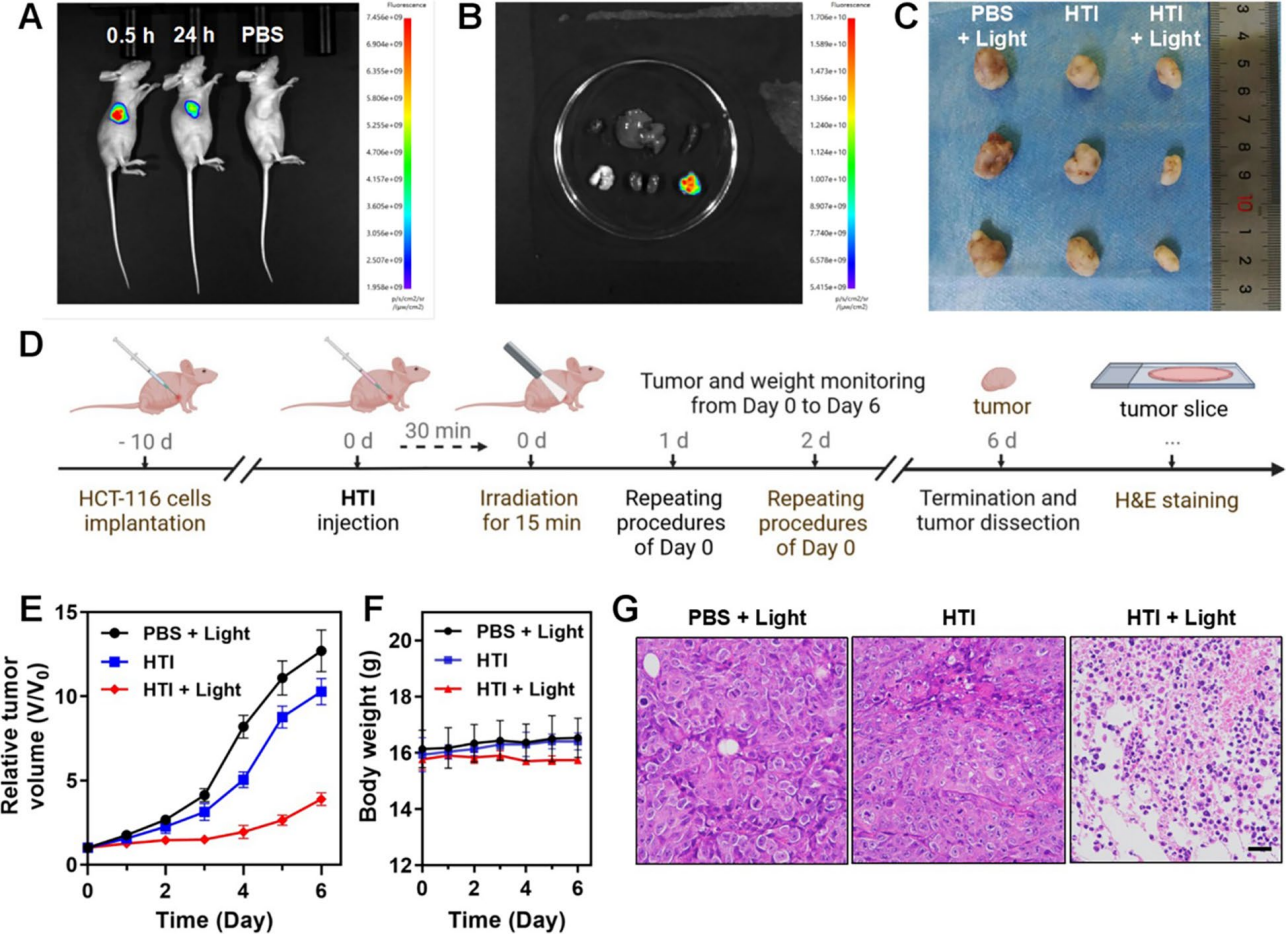
Tumor labelling and inhibition ability of HTI in early colon cancer model. **A** In vivo fluorescence image after the intratumoral injection of HTI (100 μM, 100 μL) at different times after treatment. **B** Ex vivo fluorescence image of tumor compared to normal organs (heart, liver, spleen, lung and kidney) 0.5 h after intratumoral injection of HTI. **C** Photo of the tumors harvested from tumor-bearing mice after 6 days of different treatments. **D** The schematic timeline illustration of in vivo PDT on HCT-116 tumor-bearing mice after intratumoral injection of HTI. **E** Time-dependent relative tumor volume (V/V_0_) growth line chart. **F** Body weight changes of tumor-bearing mice with different treatments. **G** H&E staining section of tumor tissue with different treatments. Scale bar, 50 μm

Boosted by the excellent performance of HTI in vitro assays, we expected to further achieve the visualization and therapy of tumor via HTI in vivo. HCT-116 cells-derived xenograft (CDX) in BALB/c nude mice was conducted. HCT-116 cells were implanted in mice 10 days before the tumor grew up to approximately 50 mm^3^ to serve as an early colorectal cancer model. In vivo fluorescence image showed the bio-compatibility and metabolic stability of HTI after the intratumoral injection of HTI (100 μM, 100 μL). As depicted in Fig. 6A, bright fluorescence signals could be captured after the injection of HTI, compared to the PBS group, owing to the high viscosity environment in tumor site. And the fluorescence signal could still be plainly captured in the tumor area after 24 h. Mice were euthanized after the treatment, then tumor together with main normal organs were isolated for ex vivo fluorescence imaging (Fig. 6B). Fluorescence was only observed in tumor which demonstrated the retention effect and the specificity of HTI towards tumor. This finely illustrated that HTI, as a feasible and efficient real-time photosensitizer with tumor retention capability, held the potential to fulfill the clinical diagnostic access to viscosity-related disease.

Then, the therapy effect of was HTI investigated in vivo. The schematic timeline was briefly illustrated in Fig. 6D. After the tumor grew up to approximately 50 mm^3^, HTI was intratumorally injected, followed by 15 min white light irradiation at the tumor site. Parallel control groups were performed with HTI without irradiation or PBS with light irradiation. Tumor and weight monitoring were conducted daily among each group. After 6 days of treatment, tumors were isolated from mice for inspecting and H&E staining before reaching the ethical limits. As shown in Fig. 6C, the growth of colorectal tumors in CDX nude mice was apparently inhibited in the ‘HTI + light’ group, compared to the parallel groups, which was elucidated in the time-dependent relative tumor volume (V/V_0_) growth (Fig. 6E). As displayed in Fig. 6F, the body weight of the three groups remained stable, indicating relatively low systemic side effects of HTI on the mice. The following H&E staining further confirmed that the tumor tissue treated with HTI and light irradiation tended to necrotize. As depicted in Fig. 6G, typical cell necrosis appearance with nuclear pyknosis based on nuclear dehydration was observed in the ‘HTI + light’ group. These results reflected that HTI was instrumental in long-term tumor inhibition and clearance. In brief, these results strongly supported HTI to be a significant viscosity-dependent photosensitizer for clinical PDT purposes on CRC.

## Discussion

Colon cancer is one of the leading cancers worldwide. The high incidence of advanced colon cancer, especially in East Asia, is a dilemma that highlights the lack of effective diagnostic methods. Generally, pathological changes are usually accompanied by fluctuations in physiological parameters from the beginning of the disease [16, 48]. Viscosity, to somewhat extent, is of attributive value for probing pathological changes in the early stage. Viscosity, conventionally considered disincentive in particle motion within fluids, found counterintuitively promoting cell migration and cancer metastasis in recent studies [49]. The elevated viscosity in extracellular matrix, owing to the accumulation of glycosaminoglycan (hyaluronic acid, etc.) and protein, increases mechanical loading for a dense actin-related network which contributes to the extension and movement of cellular fiber pseudopodia. And moreover, a peculiar mechanism of cancer cell, viscosity memory, is found capable of activating transcriptional controlled cancer-related pathway when cells are re-exposed to elevated viscosity [49, 50]. Therefore, these findings evidently demonstrate that viscosity is highly correlated with pathophysiological relevant molecular mechanisms to cancer, providing a broad research prospect for further clinical application.

Derived from these preliminary studies, increasing fluorescent probes, with unique structures and synthetic methods, were reported to be applied for viscosity detection [51–53]. As yet, probes based on intramolecular rotors are still the classic mainstream scaffolds for viscosity imaging. Nevertheless, many of them overlooked to achieving therapeutic functions which largely obstructed their potential clinical transfer and research innovation. In view of this, we aimed to propose a multifunctional fluorescent probe which was intended to consolidate mitochondrial viscosity visualization and ROS stimulation for CRC inspecting and treatment.

Herein, a novel photosensitizer HTI, benefiting from its remarkable viscosity sensitivity, small background interference, and excellent mitochondria-targeting ability, was developed for the diagnosis and treatment of CRC. Enhanced fluorescence of HTI was displayed in high viscosity environment since the intramolecular rotation was restricted. Meanwhile, the positive charge on HTI enabled it to target mitochondria through the electrostatic interaction with the mitochondrial membrane. Under these circumstances, the probe could distinguish colon cancer cells from normal cells with high spatiotemporal resolution specificity. More importantly, viscosity monitoring performance was also verified in mice models. A clear fluorescent signal could still be captured at tumor after 24 h, reflecting a good retention effect of HTI.

Then, the oncotherapy performance of HTI was investigated in vitro and in vivo. The ROS (^1^O_2_) generation capacity of HTI was proved to be stronger than Ce6 by ABDA. Based on DCFH-DA, as well as Calcein-AM, it had been demonstrated that HTI was qualified for producing ROS with high efficiency at cellular level to eliminate cancer cells. Generally, animal experiments are solid foundations between cellular biology and clinical utilization. CDX model, transplanted from human tumor cell lines which is of small tumor heterogeneity and high formation rates, is regarded as a reliable step in anti-tumor strategies. Hence, we established an early CRC model employing HCT-116 cells to conduct the validation of HTI in vivo. The tumor suppressive PDT effect initiated by HTI offering a feasible yet curative solution for inspecting and treating CRC. It represented the first validation of a multifunctional fluorescent probe in viscosity labeling and PDT effect study on CRC. Therefore, the validation we conducted in vivo model is of great significance to HTI for further clinical application.

Profiting from the significant development of synthetic organic chemistry, new fluorescent probes has been efficiently iterated [54], being able to provide sensitive and accurate bioimaging of which largely facilitate the diagnosis of diseases, including neurodegenerative disorder [55], Parkinson's disease [56], diabetes [57], inflammatory bowel disease [58], and rheumatoid arthritis [59]. Moreover, probes targeting ferroptosis have been reported, providing a visual tool for deeper exploration of disease mechanisms [60, 61]. Therefore, based on the prominent characteristics as mentioned above, HTI is destined to facilitate clinical practice of CRC in the following aspects once being properly converted. Firstly, considering the high misdiagnosis rate of early digestive tumor using standard white-light endoscopy [62], additional diagnostic methods beyond white light spectrum will be well received in clinical. HTI can appropriately provide a resolution to improve the early diagnosis rate of CRC by enhancing the sensitivity and specificity of recognizing subtle mitochondrial viscosity differences which has been validated in CRC cells and mice model. Next, complete tumor resection is evidently associated with better overall survival and lower recurrence rate [63]. HTI can also be able to assistant CRC resection by intraoperative fluorescence visualization to ensure complete resection with negative margins (R0). More importantly, photodynamic agents are promising therapeutic complements for targeted treatment of cancer [64]. HTI emerged a higher ^1^O_2_ generation performance than Ce6. The successful inhibition of tumor growth in the early CRC model demonstrated that HTI was qualified to serve as a worthy reference to the establishment of fluorescent probe-involved PDT for CRC management. Besides, the information collected from the early CRC model would profoundly inspire future relational model building and advancing.

However, there are still some defects in HTI, such as the relative high cytotoxicity and the limited fluorescence emission. Recently, the concepts of dynamic response, enzyme engineering, modular molecular engineering, nanotechnology, and logic gates are proposed in the field of biomaterials and drugs [65–68], leading to an impact on single structure and functional design. Obviously, smarter multifunctional biomaterials with stronger delivery system and barrier overcoming ability to achieve selectivity together with accuracy and mitigate the potential toxicity in vivo are anticipated in clinical practice [69]. Hence, loading the probe into a nanoparticle carrier could be a potential approach to improve its properties. Overall, we have provided an underlying tool for CRC theranostics and held great promise for other various biomedical applications.

## Conclusions

In summary, we proposed an innovative design strategy to fabricate probe HTI, which exhibited exceptional sensitivity in visualizing mitochondrial viscosity and stimulating ROS (^1^O_2_). Its high sensitiveness for mitochondrial viscosity enabled it to efficiently discriminate colon cancer cells from normal cells. Furthermore, HTI was also qualified for eliminating colon cancer cells and inhibiting tumor growth on early colon cancer model through PDT. Beyond its applications in fluorescent imaging and PDT, HTI also held intriguing potential for other medical uses, including surgery resection labeling and gastrointestinal endoscopy screening. Its versatility could also be extended to detect other diseases related to viscosity abnormalities. It could open a new avenue for early cancer detection, targeted therapy, and other potential applications in various medical procedures, contributing to improved patient care and disease management.

### Experimental section

Materials and apparatus, details of synthetic procedures can be found in the Supporting Information.

### Supplementary Information

**Additional file 1: Scheme S1.** The synthesis route of HTI. **Fig. S1.** ^1^H NMR spectrum of HTI in Methanol-*d*_4_. **Fig. S2.** ^13^C NMR spectrum of HTI in Methanol-*d*_4_. **Fig. S3.** HRMS spectrum of HTI. **Fig. S4.** Absorption spectra of HTI (10 μM) in methanol or glycerin. *λ*_*ex*_ = 500 nm. **Fig. S5.** Fluorescence intensity of HTI (10 *μ*M) in PBS buffer solutions with different pH values.*λ*_*ex*_= 500 nm. **Fig. S6.** UV−vis absorption spectra of ABDA (50 μM) with (A) or without (B) Ce6 (10 μM) upon various periods of white light irradiation (60 mW cm^−2^). **Fig. S7.** Cytotoxicity test for HTI in SW-620 cells or HCT-116 cells or NCM-460 cells. **Fig. S8.** (A) Merged channel of HCT-116 cells incubated with HTI and MitoTracker-Green. (B) Fluorescence intensities of HTI and MitoTracker-green on the white arrows in A. (C) Merged channel of HCT-116 cells incubated with HTI and LysoTracker-Green. (D) Fluorescence intensities of HTI and LysoTracker-Green on the white arrows in C. **Fig. S9.** Fluorescence emission spectra of HTI (10 μM) coexisting with nystatin (10 μM) or monensin (10 μM) in methanol. *λ*_ex_ = 500 nm.

## Data Availability

The datasets used and/or analyzed in this study are available from the corresponding author upon reasonable request.

## Abbreviations

ABDA: 9,10-Antherachenediyl-bis(methylene) dimalonic acid
CDX: Cell derived xenograft
CLSM: Confocal laser scanning microscope
CRC: Colorectal cancer
DCFH-DA: 2',7'-Dichlorodihydrofluorescein diacetate
DMSO: Dimethyl sulfoxide
HRMS: High-resolution mass spectra
HTI: 2-(2-(5-(3-(Benzo[d]thiazol-2-yl)-4-hydroxyphenyl)thiophen-2-yl)vinyl)-1,3,3-trimethyl-3H-indol-1-ium iodide
NMR: Nuclear Magnetic Resonance
PBS: Phosphate buffer solution
PDT: Photodynamic therapy
