# Supplementary material for: Precise visualization and ROS-dependent photodynamic therapy of colorectal cancer with a novel mitochondrial viscosity photosensitive fluorescent probe
Source: Biomater Res. 2023 Nov 8;27:112. doi: 10.1186/s40824-023-00450-2 (PMC10634017; doi:10.1186/s40824-023-00450-2)
Supplement: Supplementary file 1 — Additional file 1: Scheme S1. The synthesis route of HTI. Fig. S1. 1H NMR spectrum of HTI in Methanol-d4. Fig. S2. 13C NMR spectrum of HTI in Methanol-d4. Fig. S3. HRMS spectrum of HTI. Fig. S4. Absorption spectra of HTI (10 μM) in methanol or glycerin. λex = 500 nm. Fig. S5. Fluorescence intensity of HTI (10 μM) in PBS buffer solutions with different pH values.λex= 500 nm. Fig. S6. UV−vis absorption spectra of ABDA (50 μM) with (A) or without (B) Ce6 (10 μM) upon various periods of white light irradiation (60 mW cm−2). Fig. S7. Cytotoxicity test for HTI in SW-620 cells or HCT-116 cells or NCM-460 cells. Fig. S8. (A) Merged channel of HCT-116 cells incubated with HTI and MitoTracker-Green. (B) Fluorescence intensities of HTI and MitoTracker-green on the white arrows in A. (C) Merged channel of HCT-116 cells incubated with HTI and LysoTracker-Green. (D) Fluorescence intensities of HTI and LysoTracker-Green on the white arrows in C. Fig. S9. Fluorescence emission spectra of HTI (10 μM) coexisting with nystatin (10 μM) or monensin (10 μM) in methanol. λex = 500 nm. [file 40824_2023_450_MOESM1_ESM.docx]

**Precise visualization and ROS-dependent photodynamic therapy of colorectal cancer with a novel mitochondrial viscosity photosensitive fluorescent probe**

Runsha Xiao ^a,b,‡^, Fan Zheng ^c,d,‡^, Kuo Kang ^a,^, Lei Xiao ^a,e,^, Anyao Bi ^c,d,^, Yiting Chen ^f,^, Qi Zhou ^g,^, Xueping Feng ^a,g,^, Zhikang Chen ^b,^, Hao Yin ^i,^, Wei Wang ^j,k,^, Zihua Chen ^a,b,*^, Xiaomiao Cheng ^h,l,*^, and Wenbin Zeng ^c,d,*^

^‡^ These authors contributed equally to this work.

^a^ Hunan Key Laboratory of Precision Diagnosis and Treatment of Gastrointestinal Tumor, Department of Gastrointestinal Surgery, Xiangya Hospital, Central South University, Changsha, 410013, PR China.

^b^ International Joint Research Center of Minimally Invasive Endoscopic Technology Equipment &Standardization, Xiangya Hospital, Central South University, Changsha, 410013, PR China.

^c^ Xiangya School of Pharmaceutical Sciences, Central South University, Changsha, 410013, PR China.

^d^ Hunan Key Laboratory of Diagnostic and Therapeutic Drug Research for Chronic Diseases, Central South University, Changsha, 410013, PR China.

^e^ Department of Colorectal Surgery, Affiliated Cancer Hospital of Xiangya School of Medicine, Central South University, Changsha, 410013, PR China.

^f^ Department of Critical Care Medicine, Xiangya Hospital, Central South University, Changsha, 410013, PR China.

^g^ Institute of Medical Sciences, Xiangya Hospital, Central South University, Changsha, 410013, PR China.

^h^ Department of Nephrology, Xiangya Hospital, Central South University, Changsha, 410013, PR China.

^i^ Organ Transplant Center, Shanghai Changzheng Hospital, Shanghai 200003, China.

^j^ Cell Transplantation and Gene Therapy Institute, The Third Xiang Ya Hospital, Central South University, Changsha, Hunan, 410013, China.

^k^ Engineering and Technology Research Center for Xenotransplantation of Hunan Province, Changsha, Hunan, 410013, China.

^i^ Department of Nephrology, Xiangya Changde Hospital, Changde, 415000, PR China.

^*^ Corresponding authors. E-mail addresses: wbzeng@hotmail.com (W. Zeng), cxm131@126.com (X. Cheng), zihuac@outlook.com (Z. Chen).

# 1. Experimental section

## 1.1. Materials and apparatus.

All chemicals were purchased from commercial suppliers and used as received. All buffers were prepared with deionized water that had passed through a water ultra-purification system before being used. Iodomethane, 5-bromosalicylaldehyde, 2-aminobenzenethiol, 5-formyl-2-thiopheneboronic acid, and 2,3,3-trimethylindolenine were purchased from Energy Chemical Co., Ltd (Shanghai, China). Methanol (MeOH) and other organic solvents were purchased from Sinopharm Chemical Reagent Co., Ltd (Shanghai, China). Monensin and Nystatin were purchased from Topscience (Shanghai, China). ^1^H NMR (500 MHz) and ^13^C NMR (125 MHz) spectra were recorded on a Bruker Advance spectrometer (Rheinstetten, Germany) with tetramethylsilane (TMS) as an internal standard. High-resolution mass spectra (HRMS) were obtained on an Orbitrap Velos Pro LC-MS spectrometer (Thermo Scientific). UV–vis absorption spectra were measured on a UV-2450 UV-visible spectrophotometer (Shimadzu, Japan). Fluorescence spectra were recorded with a Hitachi F-2700 fluorescence spectrophotometer (Hitachi, Japan). The pH measurements were conducted with a Rex PHS-3C pH meter. Viscosity value was performed by an NDJ-8S rotary viscometer. The fluorescent cell images were acquired through a Zeiss LSM 900 confocal laser scanning microscope (Zeiss, Germany).

## 1.2. Synthesis

**Scheme** **S1**. The synthesis route of **HTI**.

Synthesis of Compound **2**.

Compound **1** (5-bromo-2-hydroxybenzaldehyde, 200 mg, 1 mmol) and 2-aminobenzenethiol (150 mg, 1.2 mmol) were dissolved in 5 mL methanol, stirring at room temperature. Five drops of 30% H_2_O_2_ and 1 drop of 1 M HCl were added to the bottle dropwise. The reaction mixture was stirred for 6 h. After the reaction was completed, the solvent was removed under reduced pressure and purified by silica column chromatography with hexane/ethyl acetate (100:1, v/v) to give Compound **2** as a pale-yellow solid (273 mg, yield: 89.6 %). ^1^H NMR (500 MHz, DMSO-*d*_6_) δ 11.70 (s, 1H), 8.38 (d, *J* = 2.6 Hz, 1H), 8.14 (d, *J* = 8.0 Hz, 1H), 8.08 (d, *J* = 8.0 Hz, 1H), 7.58 -7.52 (m, 2H), 7.49-7.42 (m, 1H), 7.06 (d, *J* = 8.7 Hz, 1H). ^13^C NMR (125 MHz, DMSO-*d*_6_) δ 162.8, 155.7, 151.8, 135.5, 134.9, 130.6, 126.9, 125.6, 122.9, 122.5, 121.4, 119.6, 111.3.

Synthesis of Compound **3**.

Compound **2** (273 mg, 0.89 mmol), 5-formylthiophen-2-boronic acid (203 mg, 1.3 mmol), K_2_CO_3_ (179 mg, 1.3 mmol), and [1,1'-Bis (diphenylphosphino) ferrocene] dichloropalladium (II) (Pd(dppf)Cl_2_, 10 mg) were added to a 100 mL three-necked bottle, then vacuumed and protected by N_2_. After that, 8 mL of methanol and 5 ml of toluene were added to dissolve the compounds, vacuuming again and protecting by N_2_. Finally, it was heated at 90 °C for 6 h. After the reaction was completed, the solvent was removed under reduced pressure and purified by silica column chromatography with hexane/ethyl acetate (40:1, v/v) to give Compound **3** as a pale-yellow solid (186 mg, yield: 55.2%). ^1^H NMR (500 MHz, CDCl_3_) δ 9.90 (s, 1H), 8.05 (d, *J* = 8.2 Hz, 1H), 8.00 (s, 1H), 7.96 (d, *J* = 7.9 Hz, 1H), 7.76 (d, *J* = 3.9 Hz, 1H), 7.69 (d, *J* = 8.9 Hz, 1H), 7.56 (t, *J* = 7.7 Hz, 1H), 7.47 (t, *J* = 7.6 Hz, 1H), 7.39 (d, *J* = 3.9 Hz, 1H), 7.20 (d, *J* = 8.7 Hz, 1H). ^13^C NMR (125 MHz, CDCl_3_) δ 182.7, 168.4, 159.1, 153.4, 151.7, 141.9, 137.6, 132.6, 130.7, 127.0, 126.2, 126.0, 124.9, 123.4, 122.4, 121.7, 118.9, 117.3.

Synthesis of **HTI**.

Compound **3** (337 mg, 1 mmol) and 1,2,3,3-tetramethyl-3H-indol-1-ium (301 mg, 1 mmol) were dissolved in 10 mL of EtOH. Piperidine (40 μL) was added dropwise, and then the mixture was reacted at 90 °C for 6 h. After the reaction was completed, the solvent was removed under reduced pressure and purified by silica column chromatography with dichloromethane/methanol (30:1, v/v) to give probe **HTI** as a dark aubergine solid (459 mg, yield: 74.1%). ^1^H NMR (500 MHz, Methanol-*d*_4_) δ 8.59 (d, *J* = 15.7 Hz, 1H), 8.30 (d, *J* = 2.3 Hz, 1H), 8.05 (dd, *J* = 10.7, 8.0 Hz, 2H), 7.94 (d, *J* = 4.1 Hz, 1H), 7.85 (dd, *J* = 8.6, 2.3 Hz, 1H), 7.76 (ddd, *J* = 6.1, 4.1, 1.6 Hz, 2H), 7.67 – 7.61 (m, 2H), 7.65 – 7.59 (m, 1H), 7.57 (t, *J* = 7.6 Hz, 1H), 7.49 (t, *J* = 7.6 Hz, 1H), 7.19 (d, *J* = 15.7 Hz, 1H), 7.17 (d, *J* = 6.1 Hz, 1H), 4.09 (s, 3H), 1.85 (s, 6H). ^13^C NMR (125 MHz, Methanol-*d*_4_) δ 181.2, 166.9, 158.7, 154.4, 151.6, 146.1, 143.2, 141.9, 139.9, 138.9, 133.5, 130.3, 129.0, 126.7, 126.0, 125.7, 125.3, 124.8, 122.4, 121.9, 121.5, 118.2, 118.1, 114.1, 109.0, 32.8, 25.1. HRMS: m/z calcd for C_30_H_25_N_2_OS_2_^+^ ([M + H]^+^) 493.1403, found 493.1397.

1.3. Anti-interference study

In the anti-interference study, various competitive analytes (Zn(OAc)_2_, FeSO_4_, CoCl_2_, CaCl_2_, MgSO_4_, NaF, NaBr, NaI, NaNO_2_, KNO_3_, NaNO_2_, Na_2_SO_3_, H_2_O_2_, Glucose, Cys, Tyr, Pro, Gly, Val, and Ala) were prepared in water.

1.4 Cell cytotoxicity

HCT-116 cells were previously seeded into 96-well plates at a density of 5×10^3^ cells/well in complete McCoy's 5A medium. **HTI** was then added into the wells at different concentrations. After 24 h of incubation, 10 μL of CCK-8 solution was added into each well and culture for 1h before determining the absorption at 450 nm using a microplate reader (Biotek, VT).

1.5 Animal experiments

Four-week-old female BALB/c nude mice, were purchased from SJA (Hunan, China), were subcutaneously inoculated with HCT-116 cells 1 × 10^6^ each, then maintained under specific pathogen-free (SPF) conditions. With the approval of the Institutional Animal Ethics Committee, experiments were conducted, following the Guide of Laboratory Animals of Xiangya Hospital, Central South University (Changsha, China).

# 2. Figures


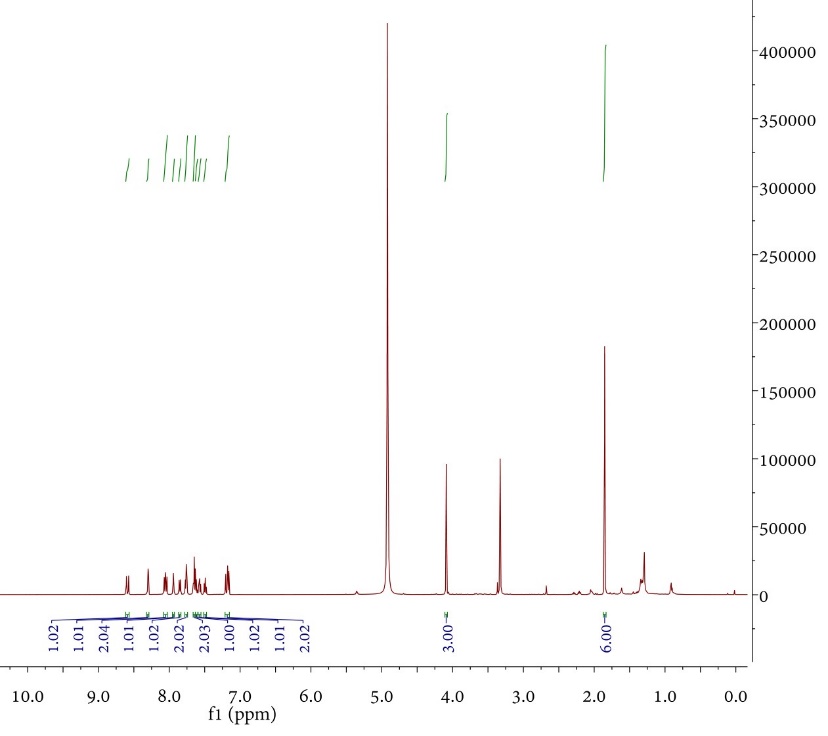


**Fig. S1.** ^1^H NMR spectrum of **HTI** in methanol-*d*_4_.


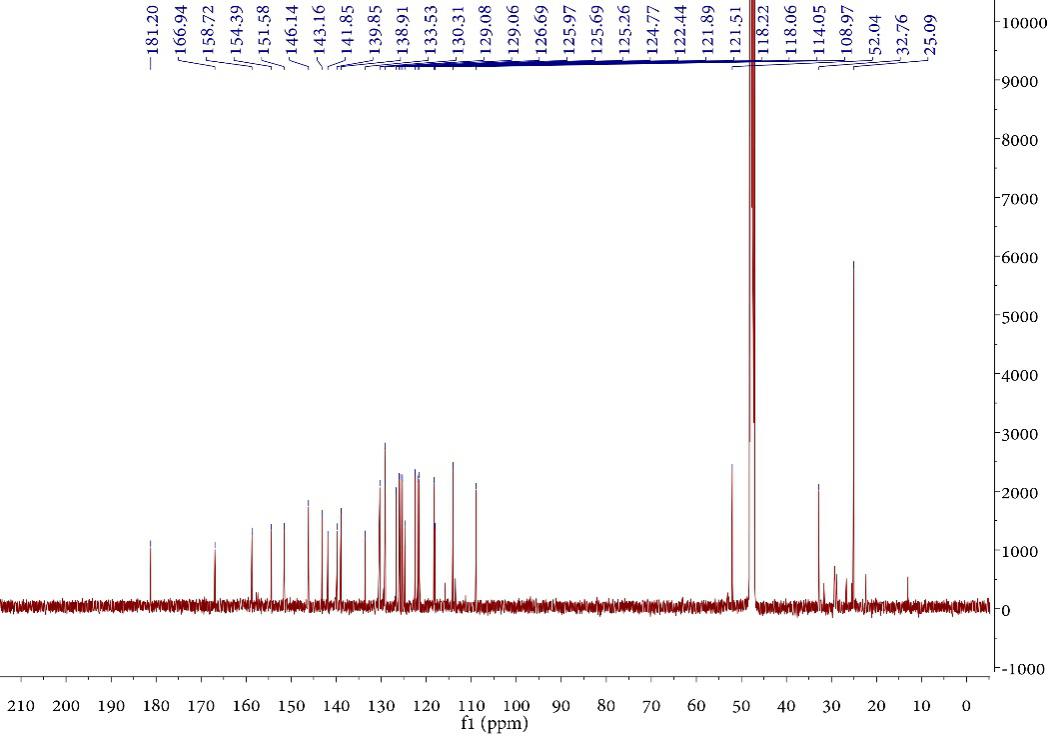


**Fig. S2.** ^13^C NMR spectrum of **HTI** in methanol-*d*_4_.

**Fig. S3.** HRMS spectrum of **HTI**.


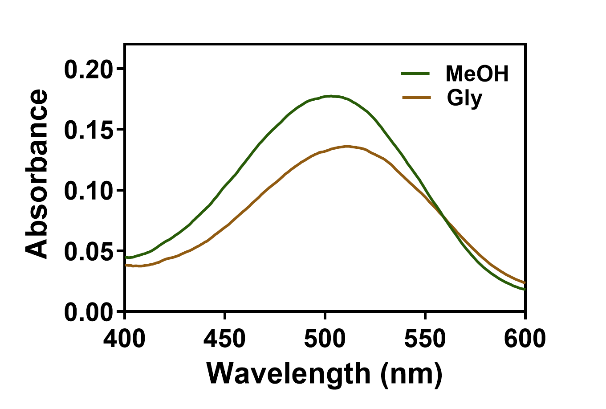


**Fig. S4.** Absorption spectra of **HTI** (10 μM) in methanol or glycerin.


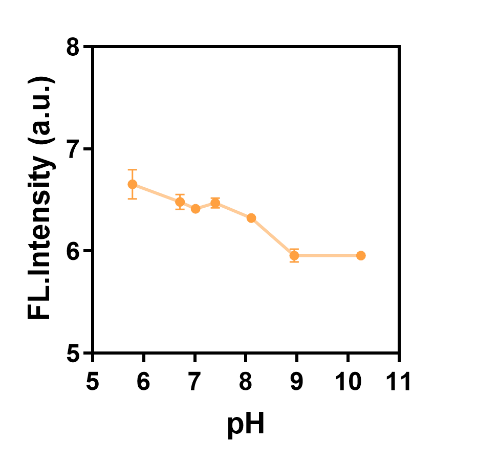


**Fig. S5.** Fluorescence intensity of **HTI** (10 *μ*M) in PBS buffer solutions with different pH values. *λ_ex_* = 500 nm.

**
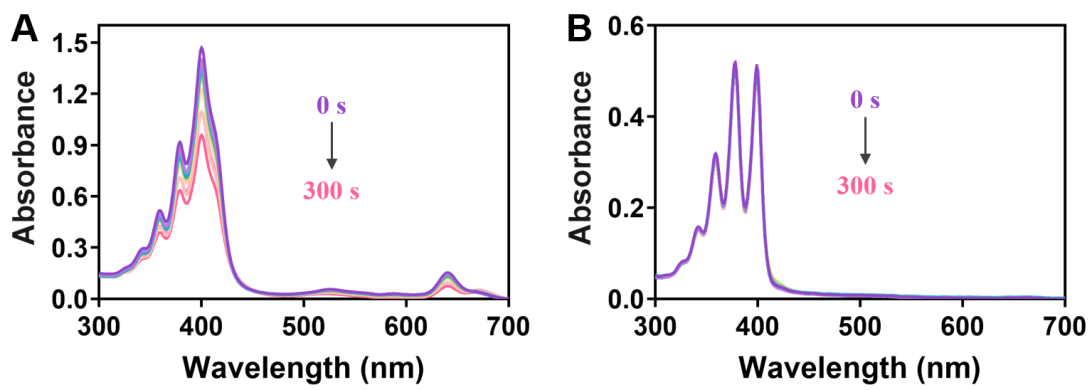
**

**Fig. S6.** UV−vis absorption spectra of ABDA (50 μM) with (**A**) or without (**B**) Ce6 (10 μM) upon various periods of white light irradiation (60 mW cm^−2^).


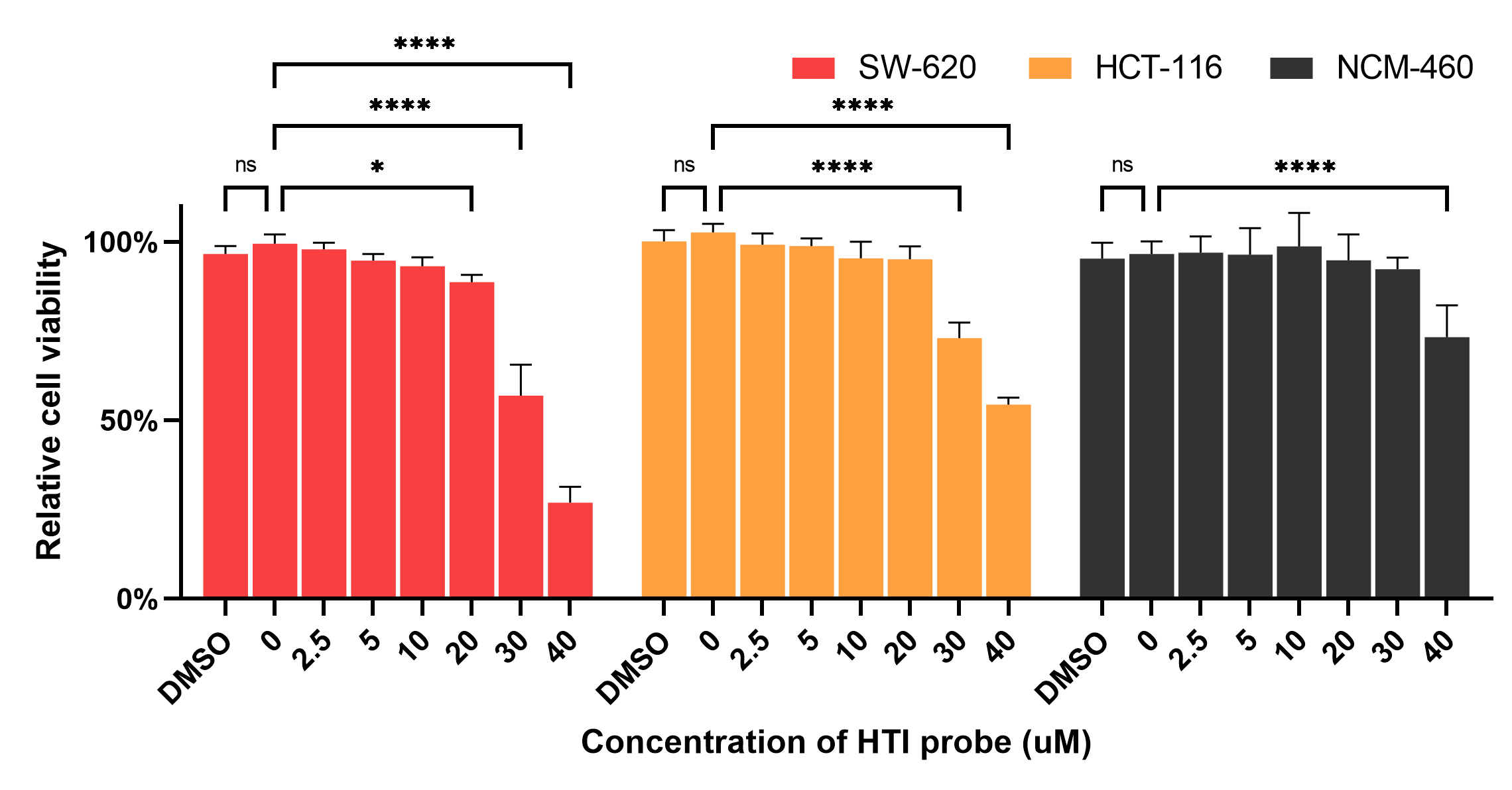


**Fig. S7.** Cytotoxicity test for **HTI** in SW-620 cells or HCT-116 cells or NCM-460 cells.


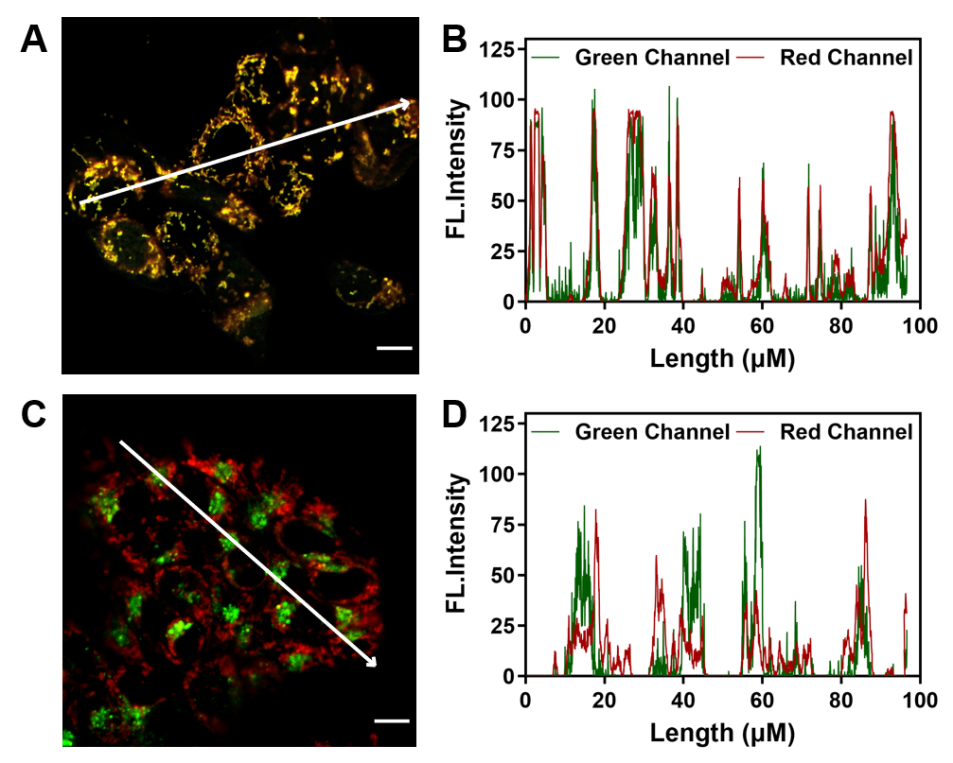


**Fig. S8.** (**A**) Merged channel of HCT-116 cells incubated with **HTI** and MitoTracker-Green. (**B**) Fluorescence intensities of **HTI** and MitoTracker-green on the white arrows in **A**. (**C**) Merged channel of HCT-116 cells incubated with **HTI** and LysoTracker-Green. (**D**) Fluorescence intensities of **HTI** and LysoTracker-Green on the white arrows in **C**.


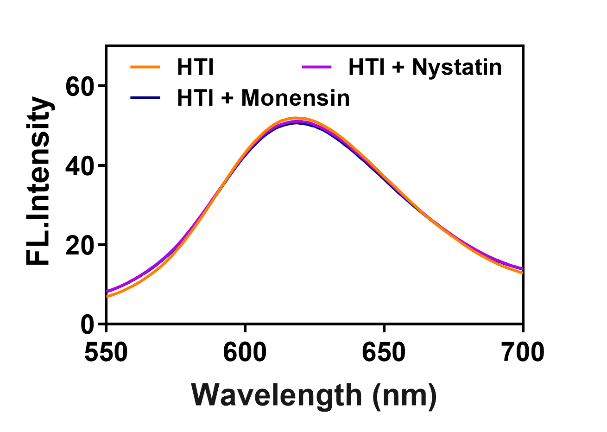


**Fig. S9.** Fluorescence emission spectra of **HTI** (10 μM) coexisting with nystatin (10 μM) or monensin (10 μM) in methanol. *λ*_ex_ = 500 nm.

# 3. Table

**Table S1**. An overview on recently reported photosensitive fluorescent probes.

| Probe | *λ*_ex_/*λ*_em_ (nm) | Stokes shift (nm) | Response | Targeting | Application | Reference |
| --- | --- | --- | --- | --- | --- | --- |
|  | 500/617 | 117 | viscosity | mitochondria | colorectal cancer  theranostics | This work |
|  | 450/625 | 175 | H_2_O_2_ | lipid droplets | breast cancer  theranostics | [1] |
|  | 565/720 | 155 | viscosity | mitochondria | cervical cancer  theranostics | [2] |
|  | 450/635 | 185 | - | lysosomes | breast cancer  theranostics | [3] |
| Ce6 (BCM) | - | - | pH and GSH | - | colorectal cancer  theranostics | [4] |
|  | n = 1: 530/625  n = 2:  630/735 | n = 1: 95  n = 2: 105 | viscosity | mitochondria | achieve cellular PDT and monitor mitochondrial viscosity during PDT | [5] |
|  | 556/660 | 104 | viscosity | lysosomes | achieve cellular PDT | [6] |
|  | 520/660 | 140 | - | - | lung cancer  theranostics | [7] |

Reference:

1. Jiang G, Li C, Liu X, Chen Q, Li X, Gu X, et al. Lipid droplet‐targetable fluorescence guided photodynamic therapy of cancer cells with an activatable AIE‐active fluorescent probe for hydrogen peroxide. Adv Opt Mater. 2020;8:2001119.

2. Fan L, Zan Q, Wang X, Yu X, Wang S, Zhang Y, et al. A mitochondria-targeted and viscosity-sensitive near-infrared fluorescent probe for visualization of fatty liver, inflammation and photodynamic cancer therapy. Chem Eng J. 2022;449:137762.

3. Huang L, Qing D, Zhao S, Wu X, Yang K, Ren X, et al. Acceptor-donor-acceptor structured deep-red AIE photosensitizer: Lysosome-specific targeting, in vivo long-term imaging, and effective photodynamic therapy. Chem Eng J. 2022;430:132638.

4. Liang X, Mu M, Chen B, Chuan D, Zhao N, Fan R, et al. BSA-assisted synthesis of nanoreactors with dual pH and glutathione responses for ferroptosis and photodynamic synergistic therapy of colorectal cancer. Mater Today Adv. 2022;16:100308.

5. Yang L, Chen Q, Wan Y, Gan S, Li S, Lee CS, et al. A NIR molecular rotor photosensitizer for efficient PDT and synchronous mitochondrial viscosity imaging. Chem Commun. 2022;58:9425-8.

6. Pan Z, Wang Y, Chen N, Cao G, Zeng Y, Dong J, et al. Aggregation-induced emission photosensitizer with lysosomal response for photodynamic therapy against cancer. Bioorg Chem. 2023;132:106349.

7. Shen H, Li Y, Kang X, Wu J, Chen R, Wei X, et al. Cancer-cell-biomimetic carbazole-based AIE nanoplatform for targeted phototheranostics of lung cancer. ACS Appl Nano Mater. 2023;6:6056-65.
